# Flu-Phobia: Influenza Vaccine Hesitancy in a Rural Primary Care Setting

**DOI:** 10.7759/cureus.72043

**Published:** 2024-10-21

**Authors:** Samantha M Lavertue, Richard Terry, Rachael Muggleton

**Affiliations:** 1 Family and Community Medicine, Lake Erie College of Osteopathic Medicine, Elmira, USA; 2 Pediatrics, Albany Medical Center, Albany, USA; 3 Family Medicine, Lake Erie College of Osteopathic Medicine, Elmira, USA; 4 Family Medicine, Arnot Ogden Medical Center, Elmira, USA

**Keywords:** covid-19, influenza, influenza vaccine, rural primary care, vaccination, vaccine hesitancy

## Abstract

The sentiments expressed by individuals, such as “I do not trust vaccines,” “I do not believe in vaccines,” and “I do not want a shot,” are commonly encountered by physicians during the annual influenza season. This study investigates vaccine hesitancy regarding the influenza vaccine within a rural primary care setting in New York State. Observations of subjective comments from community members prompted an inquiry into whether the contentious discourse surrounding the COVID-19 vaccine has influenced patients' decisions about the influenza vaccination. We hypothesized that over 50% of patients would decline the influenza vaccine during the 2023-2024 season and that many would attribute their refusal to the controversies associated with the COVID-19 vaccine. The findings of the study revealed that while more than 50% of surveyed patients did indeed refuse the influenza vaccine, only a minority linked their decision to the COVID-19 vaccine controversy. This paper examines the attitudes of a rural community toward the influenza vaccine and proposes potential strategies to address future vaccination disparities.

## Introduction

Influenza viruses A and B belong to the family *Orthomyxoviridae* and are characterized by segmented, negative-strand RNA genomes [1]. Features of influenza viruses include high mutation rates and antigenic variability, which makes efforts to decrease infection rates difficult [2]. The deadly history of influenza is long and storied, with the earliest documented pandemic dating back to 1580. Since that time, numerous other pandemics and seasonal outbreaks have occurred worldwide. Between 2010 and 2023, estimates of influenza cases range from 9.3 million to 41 million illnesses, 100,000 to 710,000 hospitalizations, and 4,900 to 51,000 deaths annually [3].

The replication cycle of influenza viruses is rapid compared to many other viruses, with new viral particles being released within hours of viral entry into host cells [4]. Reasons for this fact include that RNA-based viruses replicate faster than DNA-based viruses, and single-stranded viruses also replicate faster than double-stranded viruses [2,4]. This swift replication after just one infection is one reason why influenza spreads so efficiently and can cause widespread outbreaks [4]. It is estimated that after every century, there are three to four new influenza strains so different from the previous ones that they are capable of causing pandemics [5].

Since the first flu vaccine was developed in 1945, vaccination has become a crucial tool in preventing flu infections and reducing patient deaths worldwide. The World Health Organization (WHO) reviews flu vaccine strains biannually to match the predicted circulating strains for the following season. Researchers use phylogenetic trees to predict which strains will likely grow during the upcoming flu season. The CDC selects flu viruses for the seasonal flu vaccine based on a variety of data, including genetic data and epidemiologic data [6,7]. By updating the vaccine annually, it remains effective against the evolving flu viruses, helping to reduce the impact of flu-related illness and complications [7].

According to the CDC, the 2023-2024 flu vaccine is effective at reducing the risk of getting the flu. The vaccine's effectiveness (VE) against influenza A ranged from 46% to 59% for children and adolescents and from 27% to 46% for adults. VE against influenza B ranged from 64% to 89% for pediatric patients in outpatient settings and from 60% to 78% for all adults. The CDC also states that the vaccine makes people 27-46% less likely to visit a healthcare provider due to the flu and 40-42% less likely to be hospitalized. These findings demonstrate that the 2023-24 seasonal influenza vaccine is effective at reducing the risk of illness that would require medical attention [8]. A recent study estimated that flu vaccination reduced the risk of flu-related emergency department and urgent care visits by almost half and hospitalizations by more than a third among US adults during the 2022-2023 season [8,9]. The flu vaccination is the best way to prevent unnecessary deaths from the flu. The CDC’s Advisory Committee on Immunization Practices (ACIP) recommends annual flu vaccination for all persons six months of age or older. Contraindications for all vaccine types are limited to severe allergies with more stringent contraindications for live-attenuated vaccine types [10].

Vaccine hesitancy has emerged in the last several years as an area of considerable concern in epidemiology. Vaccine hesitancy refers to the delay or refusal of vaccination despite the availability of vaccines. It encompasses a range of factors, including complacency, lack of confidence, and convenience. Individuals may hesitate to vaccinate due to concerns about vaccine safety, efficacy, or religious or philosophical beliefs. Vaccine hesitancy poses a growing challenge to immunization programs worldwide, as it can lead to outbreaks of vaccine-preventable diseases and hinder efforts to achieve herd immunity. Addressing vaccine hesitancy requires understanding and addressing the underlying reasons for reluctance, promoting accurate information about vaccines, and building trust in immunization programs and healthcare systems [11]. This study aims to identify local perspectives on vaccination and the psychosocial barriers to patient vaccination. Our goal is to improve community flu vaccination rates and reduce the annual disease burden in rural areas by raising awareness about the reasons for vaccination hesitancy and emphasizing the importance of educating patients on flu vaccinations.

## Materials and methods

This survey was conducted at Eastside Primary Care in Elmira, NY between December 15, 2023, and March 15, 2024. This primary care office has a patient population consisting largely of medically underserved patients. The survey questions were written in a format that was easy to understand and interpret regardless of age and education level to get accurate responses. Any patient over the age of 18 was eligible to complete the survey. For patients who were unable to read or write, assistance was available for survey completion. Surveys were handed to each unique patient by the secretarial staff during their check-in process. The patients were told this was a voluntary and anonymous survey. Surveys were completed on paper with pen and without the use of protected health information and then placed into blank envelopes that were further placed within a marked box. Researchers were not present at the time when surveys were conducted and thus are blind to the identities of all survey participants. The box was then collected on the Friday of each week and each new survey was added to the total pile until the conclusion of the study. This study was granted IRB exemption from the Lake Erie College of Osteopathic Medicine Institutional Review Board on the grounds that no identifiable patient information was recorded or stored while the study took place. Once all forms were collected and the collection window ended, Google Forms (Google, Mountain View, CA) was used to digitally input the physical surveys, and Google Sheets (Google) was utilized for data analysis.

The questionnaire for this survey was designed specifically for this study and approved by the institutional review board. This survey can be viewed in the Appendices in table format. Participants were informed as to the purpose of the study, the handling of their information, and their rights to refuse the survey or end it at any time. Patients were asked whether they were offered a flu vaccine this season and whether they took the offered vaccine. Following these questions, they were given options to select for reasons they might not want to take a flu vaccine if they had refused. They were then asked whether knowledge regarding the effectiveness of a vaccine in particular or the previous year would affect their choice to take a vaccine or not. Finally, they were asked if the controversy surrounding COVID-19 vaccines played any role in their decisions regarding the flu vaccine. Yes or no questions were indicated as such, each question was to have only one answer chosen, which was explained on the survey, and reasoning questions were provided with select all functionality. The final question regarding COVID-19 was equipped with yes or no boxes in addition to a free text area where patients could explain any choice they made regarding the effect of COVID-19 on their decision to vaccinate.

## Results

**Figure 1:**
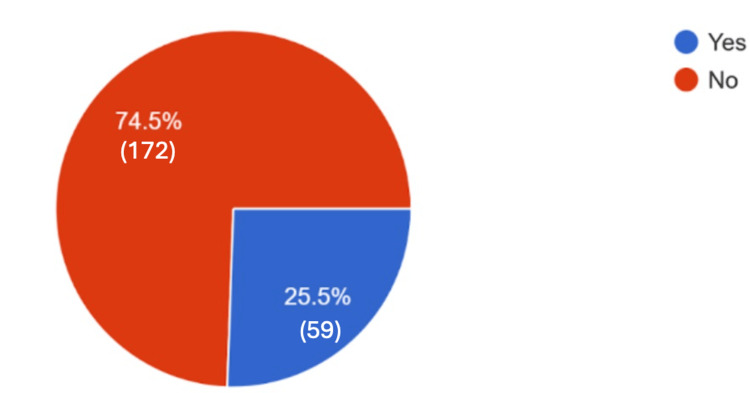
Responses to whether respondents took the influenza vaccine. Responses to the question, "Did you take the flu vaccine?". A total of 231 responses.

This survey received a total of 231 responses in the period of 12/15/2023-3/15/2024. Of these respondents, 74.5% reported they refused the flu vaccine this season, as can be seen in Figure 1.

**Figure 2:**
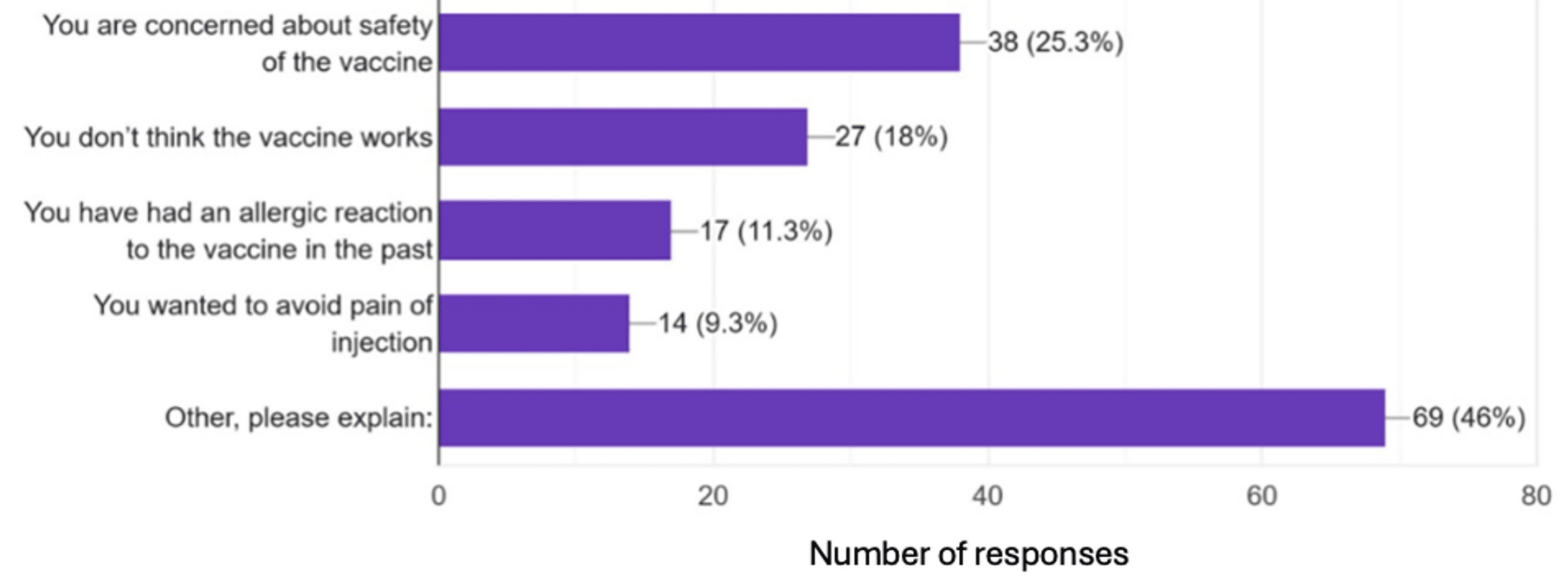
Reasoning behind the refusal of the influenza vaccine. Responses to the question, "If you said no to the vaccine, was it because ____?". A total of 150 responses.

Figure 2 below shows that 150 patients indicated preset reasoning for their refusal of the vaccine, and 46% of these patients indicated other reasoning for their refusal. A large portion of the patients who selected “other” did not end up writing anything to indicate why they had selected the other box despite being asked to explain their reason if it was not one of the others listed. The most common reason listed for “other” was that they would not take the vaccine again because it had made them “sick” the last time they had received the flu vaccine, and ultimately 8.22% of patients stated they had no interest in a flu vaccine because it makes them sick after each dose. Another commonly cited reason for refusing the vaccine was that patients had received the vaccine and then gotten the flu anyway, so they felt that getting the vaccine was useless.

**Figure 3:**
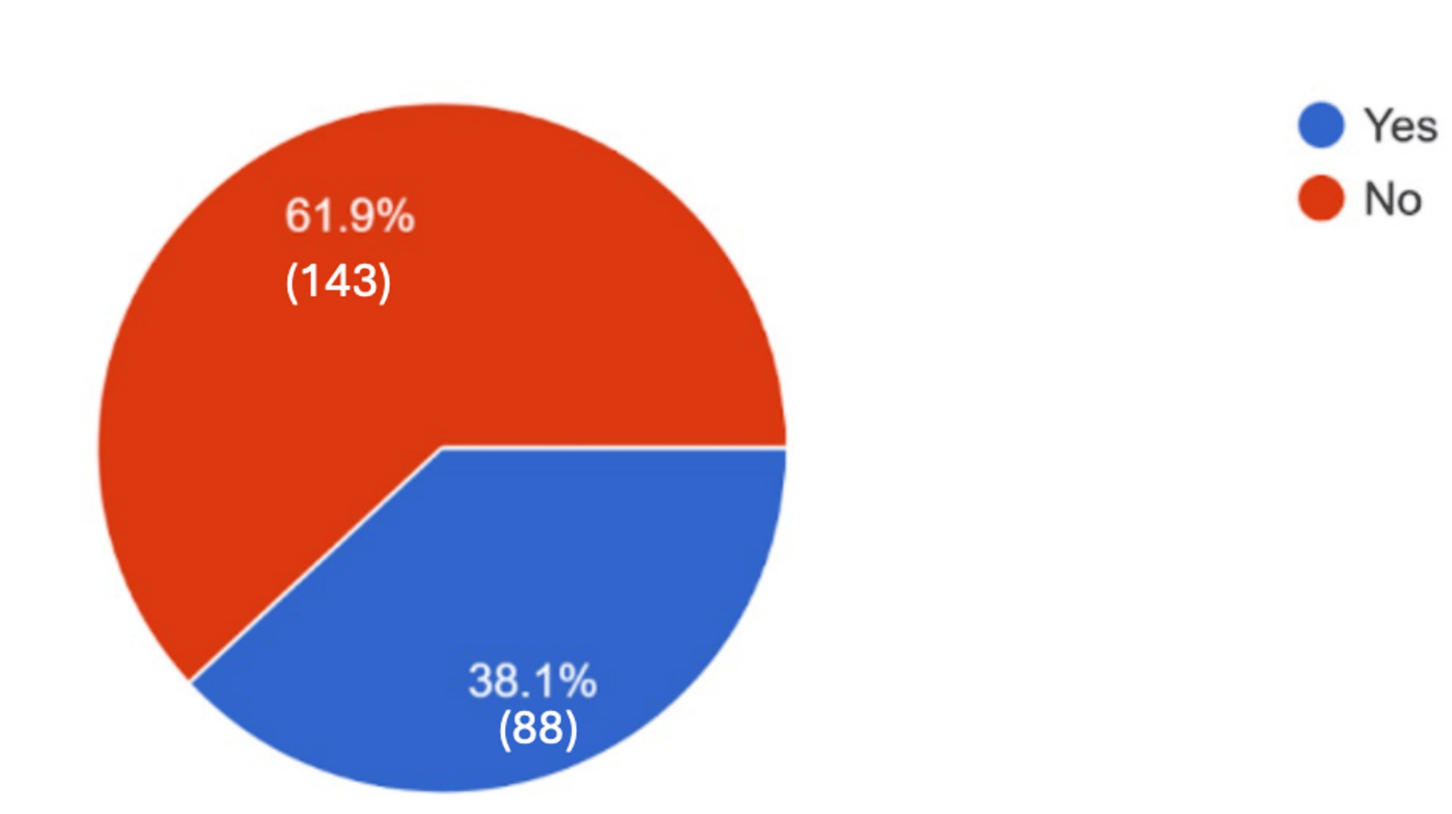
Responses regarding the effectiveness of vaccines in the decision-making process. Responses to the question, "Does knowing whether the vaccine is effective influence your decision to take it?". A total of 231 responses.

When asked whether knowing a vaccine was effective would affect their decision to vaccinate, 61.9% said no, as can be seen in Figure 3.

Due to the free-text nature of the final question regarding the COVID-19 vaccine controversy’s effect on the choice to vaccinate against flu, the results are summarized in Table 1 below according to the relative frequency in which the same reasoning was cited. One-off responses or responses that were inappropriate or off-topic have been removed for clarity.

**Table 1:** Has controversy surrounding COVID-19 vaccination and boosters changed your willingness or likelihood of receiving a flu vaccine? Explain why yes or why no. The data are presented in a "number of responses (percentage of responses out of the 231 total responses)" format. A total of 27 (11.7%) participants left this section unanswered. ** Of the 23 patients who reported “No,” no relation to COVID-19, did not get vaccination due to personal health & safety concerns, 19 (82.6%) of them stated it was due to them feeling sick afterward.

Response	Total (231 responses)
Yes	
No explanation given	9 (3.9%)
Due to concerns about effectiveness and lack of appropriate research	12 (5.2%)
Due to personal health & safety concerns	13 (5.6%)
Reasons other than lack of effectiveness and research or health safety concerns	10 (4.3%)
No	
No explanation given	56 (24.2%)
I just don’t want it	44 (19%)
No relation to COVID-19, did not get vaccination due to personal health & safety concerns**	23 (9.96%)
Reports to believe in flu shot effectiveness or receive their flu shot annually	37 (16%)

## Discussion

At the outset of this study, we hypothesized that more than 50% of patients would refuse the influenza vaccine in the 2023-2024 season and that many would cite the COVID-19 controversy as one of their reasons for refusing vaccination. Our hypothesis was proven correct in that 74.5% of respondents refused flu vaccination at the time of their survey; however, only 19% reported that the COVID-19 controversy had anything to do with their decision.

Table 1 shows a more in-depth explanation of flu shot hesitancy and compliance in Chemung County. Of the participants, 53% stated that their hesitancy to the influenza vaccination has nothing to do with the controversy surrounding the COVID-19 vaccination. Most of the hesitancy in their group comes from concerns of side effects or potentially getting sick following the vaccination. For those who associated their flu vaccination hesitancy with the COVID-19 vaccination controversy, their reasons seemed to split evenly between lack of research, lack of efficiency, and fear of side effects. These reasons for hesitancy may highlight the importance of patient education regarding vaccinations and how vaccine education must be an integral part of a physician's practice.

Vaccine hesitancy refers to a reluctance or refusal to accept vaccines despite their availability. Various models have been proposed to understand it, such as the “Three C’s” model by MacDonald, which highlights complacency, convenience, and confidence as key factors, and the Vaccine Hesitancy Continuum by the Sage Working Group, which describes a range from complete acceptance to total refusal of vaccines. The impact of vaccine hesitancy includes reduced vaccine coverage, which can lead to disease outbreaks and strain on healthcare systems [12].

Vaccines have been crucial in combating infectious diseases like smallpox, polio, and measles, highlighting early recognition of vaccines' life-saving potential. Despite their success, vaccines have faced historical resistance, often due to misinformation and fear, which has impeded their benefits. This resistance continued through various public health measures, such as mandatory vaccination laws, and evolved with new controversies, including the discredited 1998 study linking the measles, mumps, and rubella (MMR) vaccine to autism. Despite substantial evidence proving the safety of vaccines, misinformation continues to fuel skepticism, particularly amplified by digital media [12]. The factors underlying vaccine hesitancy are multifaceted and extend beyond a mere deficit in knowledge. Family physicians, as trusted sources of vaccine-related information, are instrumental in fostering vaccine acceptance [13].

Anti-vaccine sentiments have existed since early vaccination efforts. Historical examples include objections to smallpox vaccination due to fears about altering nature. Effective communication from trusted authorities, including scientific experts and community leaders, is crucial in countering vaccine hesitancy. Providing clear, evidence-based information and addressing safety concerns can help build public trust [14].

In a previous study conducted by these researchers in 2021, in the same clinic regarding COVID-19 vaccine hesitancy, views on COVID-19 vaccination were passionate: several patients reported that no amount of information about the vaccine would change their minds about receiving it. Many patients left comments about wanting a vaccine that lasted longer than a year or how one can still catch COVID-19 despite vaccination. The results from that study highlighted a local distrust of such frequent vaccinations and a profound lack of understanding of how vaccines work [15]. Our study assumed that there was opposition to the flu vaccine due to concerns about COVID-19 for various reasons; however, our data suggest that concerns about the COVID-19 vaccine had little to do with opposition to the flu vaccine and more to do with features of vaccines themselves.

The COVID-19 pandemic has highlighted the importance of widespread vaccination to control the virus. However, vaccine hesitancy has been a significant barrier. A study done in 2023 investigated the acceptance of the COVID-19 vaccine and overall factors relating to why people are more likely to be accepting. Factors associated with higher vaccine acceptance included being male, married, educated, having a history of flu vaccination, having higher income, having comorbidities, and living in urban areas [16]. Elderly individuals and those with chronic illnesses were more likely to accept the flu vaccine. Increased vaccine acceptance was also seen among those with higher body mass indexes [17]. Although these specific identifiers were not known due to the anonymity of our study, it does give insight into factors other than vaccine fatigue that may influence a patient's acceptance of a vaccine. Regardless of factors, enhancing public awareness about the benefits of vaccines is essential for overcoming vaccine hesitancy [16].

Flu vaccine uptake was high during the 2020-2021 flu season but declined in the subsequent winter. This decline was attributed to reduced flu virus activity, overshadowed by COVID-19, and growing vaccine mistrust. It has been shown that people who perceived themselves at higher risk were more likely to get the influenza vaccine. On the contrary, those who refused, explain they often felt protected without needing the vaccine [17].

Of patients who indicated a negative response to the final questions, 19% of patients stated additionally that they “just don’t want it” without any reasoning as to why they are opposed to vaccinating against influenza. This data point serves as a complicating factor to future recommendations. Ultimately, a researcher can do very little with the response, “I just don’t want it.” With COVID-19 vaccination, the medical community battled bad media coverage, “fake news,” governmental interference, and a lack of well-documented studies and good educational materials for promoting a vaccine that was so newly developed. There were targetable metrics: get more validated studies, have a better idea of adverse effects, and approach patients in a way that limits political discussion in favor of taking a personal approach to health [15]. Regarding, this population’s opinion on flu vaccination, our options for targeting vaccination gaps are limited in the face of a simple indifference to vaccination. Vaccine hesitancy is complex and influenced by various factors, including media coverage, health system trust, and pandemic conditions [17].

“Vaccine fatigue” is a topic here to consider. Vaccine fatigue is defined as “an unwillingness or inaction toward vaccine information or instruction due to perceived burden or burnout [18].” Though this topic gained in popularity in relation to COVID-19, the term pre-dates the pandemic and mostly cites its origins in the need for yearly vaccinations against the flu. Predisposing factors to vaccine fatigue include the number of required boosters in a short period of time, limited efficacy, bad science, and high rates of adverse effects [18]. While the flu vaccine requires an annual booster, the science behind the vaccine and its history of decreasing rates of death and hospitalization from influenza are well documented [3,6-10]. Ultimately the data do not suggest that COVID-19 played much of a role regarding our patients’ opinions on influenza; however, given what we know about vaccine fatigue and the number of doses required for COVID-19 in the last four years plus annual flu vaccination, we suspect it might still be playing a role in this population that this study did not capture. Further examination into vaccine fatigue may provide benefits for future studies.

One targetable area for conversation in the doctor’s office is adverse effects. While nothing can be done to decrease the number of adverse effects of flu vaccine administration, how we talk about adverse effects can be changed. Going back to Table 1, 5.6% of survey respondents indicated that they had personal health and safety concerns regarding the flu vaccine, and many of these respondents (82.6%) further indicated on their survey form things like, “the flu shot always makes me sick” or “the flu shot gives me the flu.” These comments relate to a lack of education on how vaccines work and the normal symptoms to expect after receiving a vaccine, certainly a targetable metric in an office visit.

The limitations of this study are as follows: a total of 231 responses is a large number of responses for this type of study, but this number of responses may not be extractable to the full community. While this clinic is a rural community clinic, the population contains many Medicare and Medicaid patients, which may skew results toward a smaller subset of the population. Referencing the results in Table 1, it seemed that patients may have misunderstood the question that was asked and that the rates of a positive response should have been higher, but ultimately, only 19% of respondents actually confirmed that controversy regarding COVID-19 affected their choice to vaccinate for flu. Comments such as the vaccine making people sick are misleading in the dataset, but they do highlight a need for conversations about the typical side effects of vaccines.

## Conclusions

In this study, we hypothesized that more than 50% of patients would refuse the influenza vaccine in the 2023-2024 season and that many would cite the COVID-19 controversy as one of their reasons for refusing vaccination. With 74.5% refusing vaccination, our hypothesis was partially correct; however, with only 19% reporting that the COVID-19 controversy had anything to do with their decision, the latter half of our hypothesis was incorrect. We believe that the COVID-19 vaccine controversy may have still played a role in the flu vaccine refusal; however, our data do not provide enough evidence to come to that conclusion. Ultimately, we are left with the following questions for future examination: Would changing the way we discuss vaccines in an office visit encourage more patients to vaccinate with an understanding of how vaccines actually work? How can physicians approach patients who, in spite of all the good evidence, simply do not want to vaccinate? The fact remains that some topics around vaccination are targetable and some are not. Community doctors cannot make the vaccine have fewer adverse effects, and we cannot recommend a less than adequate vaccination schedule, but we can reframe how we talk about adverse effects and the vaccine’s efficacy in preventing serious infection. More studies will need to be done to determine whether the solution to this gap in vaccination is actually a conversation-targetable problem or whether we are left to deal with the consequences of an ever-indifferent population, unconcerned with the consequences of action in the face of a right-now decision.

## Appendices

Patient survey: Flu vaccine

Dr. Terry is collecting information about patients’ opinions on the flu vaccine this year. Participation is not required, and you may stop at any time. This survey should take you less than two minutes to complete. Participation gives us permission to use responses in future publications. Please do not record any of your personal information on this piece of paper so that your responses remain anonymous. Thank you for helping our research by sharing your opinions!

**Table 2:** Survey given to participants in this research.

Patient survey: Flu vaccine
1. Were you offered the flu vaccine this year? (Circle one)
a. Yes
b. No
2. Did you take the flu vaccine? (Circle one)
a. Yes
b. No
3. If you said no to the vaccine, was it because (Circle all that apply. Do not answer this question if you took the vaccine)
a. You are concerned about the safety of the vaccine?
b. You don’t think the vaccine works?
c. You have had an allergic reaction to the vaccine in the past?
d. You wanted to avoid the pain of injection?
e. Other reasons?
i. Please explain:
4. Does knowing whether the vaccine is effective influence your decision to receive it? (Circle one)
a. Yes
b. No
5. If you circled no, please skip this question. If you circled yes, does knowing that the vaccine last year was very effective influence your decision to get the flu vaccine this year? (Circle one)
a. Yes
b. No
6. Has controversy surrounding COVID-19 vaccination and boosters changed your willingness or likelihood of receiving a flu vaccine? (Circle and explain. You may use the back of this sheet to explain in more detail.)
a. Yes, because _________________________________
b. No, because _________________________________
